# Pressure RElieving Support SUrfaces: a Randomised Evaluation 2 (PRESSURE 2) photographic validation sub-study: study protocol for a randomised controlled trial

**DOI:** 10.1186/s13063-017-1851-5

**Published:** 2017-03-20

**Authors:** Elizabeth McGinnis, Sarah Brown, Howard Collier, Phil Faulks, Rachael Gilberts, Clare Greenwood, Valerie Henderson, Delia Muir, Andrea Nelson, Jane Nixon, Isabelle Smith, Nikki Stubbs, Kay Walker, Lyn Wilson, Susanne Coleman

**Affiliations:** 1 Clinical Trials Research Unit, Leeds Institute of Clinical Trials Research, University of Leeds, Leeds, LS2 9JT UK; 2 Medical Teaching Centre, University of Leeds, Leeds, LS2 9JT UK; 3 Leeds Teaching Hospitals NHS Trust, Leeds, UK; 4 Leeds Community Healthcare NHS Trust St Marys Hospital, Leeds, LS12 3QE UK; 5 School of Healthcare, University of Leeds, Leeds, LS2 9JT UK; 6 Mid Yorkshire Hospitals NHS Trust, Wakefield, UK; 7 Pressure Ulcer Research Service User Network, Leeds, UK; 8 Northumbria Healthcare NHS Foundation Trust, North Tyneside, UK

**Keywords:** Pressure ulcer, Wound photography, Blinded outcome assessment, Randomised controlled trial

## Abstract

**Background:**

PRESSURE 2 is a randomised evaluation of the clinical and cost effectiveness of two types of pressure relieving mattress for the prevention of pressure ulcers. The primary endpoint is the time to development of a Category ≥2 pressure ulcer. The current ‘gold standard’ for the identification of a Category ≥2 pressure ulcer is expert clinical assessment. Due to the appearance of the bed, it is not possible to achieve blinding of the endpoint. This therefore poses a risk to the internal validity of the study.

A possible approach is to use photographs of skin sites, with central blinded review. However, there are practical and scientific concerns including whether patients would agree to photographs; the burden of data collection; the quality of photographs; the completeness of data; and how the use of photographs compares with the current ‘gold standard’. This validation sub-study aims to assess and quantify potential bias in the reporting of the trial endpoint.

**Methods/design:**

Patients will be specifically asked to consent to photographs being taken of their skin sites. Photographs will be taken at first observation or when patients develop a new Category ≥2 pressure ulcer (to assess over-reporting). A 10% random sample of patients will be identified for additional photographs of two skin sites (one torso and one limb) with and without a pressure ulcer (if present) by an independent assessor (to assess the potential for under-reporting).

Staff will be trained to take photographs using a standardised camera and photographic technique. A ‘grey scale’ will be included in the photo to correct white balance. Photographs will be securely transferred for central review. Photographs will have white balance corrected, and the computer monitor will be calibrated prior to review.

Analysis will include assessment of under- and over-reporting, acceptability of photography to patients, secure transfer of data, quality of and confidence in blinded photograph review and sensitivity analysis using photograph assessment of primary outcome.

**Discussion:**

This study will use photographs to contribute to the primary outcome of the trial. It will inform our understanding of the acceptability of photography for prevention trials and the possibility of other uses of photographic data in clinical work and research.

**Trial registration:**

ISRCTN, ISRCTN01151335. Registered on 14 May 2013.

**Electronic supplementary material:**

The online version of this article (doi:10.1186/s13063-017-1851-5) contains supplementary material, which is available to authorized users.

## Background

PRESSURE 2 is a randomised evaluation of the clinical and cost effectiveness of two types of pressure relieving mattresses, an alternating pressure mattress (APM) versus a high specification foam (HSF) mattress, for the prevention of pressure ulcers (PUs) [1]. The primary endpoint is the time to development of a new Category ≥2 PU [2]; secondary objectives include comparing the time to developing a new Category 1 PU between each mattress and cost effectiveness.

Expert clinical examination of the skin areas at risk of PU development is the current ‘gold standard’ for the diagnosis of the presence or absence of a PU [3, 4]. Due to the physical differences and mode of action between the APM and the HSF mattress, using the ‘gold standard’ of expert clinical assessment means that blinding of the endpoint is not achievable. In a mattress trial it is not possible to blind participants, the clinical team or the clinical research nurse (as it is obvious if a patient is on either an APM or a HSF mattress — due to the presence of a pump, the sound of the pump and the appearance of the bed and sheeting). Moving the patient to a different mattress to assess the skin is neither ethically appropriate, due to illness and frailty, nor practical, in terms of nurses’ time.

This inability to perform blinded outcome assessment poses a risk to internal validity if research nurses are influenced in some way by the mattress, with biased under- or over-reporting of the primary endpoint (Category ≥2 PU). That is, it is possible that the trial primary endpoint could be misreported by clinical research staff, if there are explicit or covert preferences for one mattress over the other. For example, this might lead to the reporting of an area of skin damage as being secondary to incontinence, or being classed as not severe enough to be a Category 2 PU, if a nurse feels that the patient is on the ‘best’ mattress and that any damage is ‘not likely’ to be a PU. There are also potential problems with the gold standard assessment because it is based on the clinical appearance of the skin. There is inherent subjectivity, and misclassification of the skin may also occur [3–5]. If a trial has a potentially biased primary outcome measure, then the findings of the trial may not be reliable, as there is a risk that estimates of the treatment effect will be biased. Therefore, it is important to assess and quantify any potential bias through estimates of over-reporting and under-reporting of Category 2 PUs.

A potential approach would be to take photographs of skin sites and assess them centrally (blind to treatment allocation), but to date this method has not been utilised in a prevention trial, and there are both practical and scientific concerns regarding its use to assess the primary endpoint: that photography would provide a high data acquisition burden to patients, it would require two nurses to position and photograph each patient and its use would raise concerns about patient consent. There are also potential concerns about image quality in ambient lighting conditions and the reliability of the identification of a Category 2 PU using photographic evidence [4, 5]. Further scientific and practical issues include:

1. Determining how the use of photographs compares to the current ‘gold standard’ of expert nurse clinical assessment in accurate diagnosis of a Category 2 PU
2. Issues regarding the aim of blinding in preventing both differential under-reporting (false negatives) and over-reporting (false positives) of PUs:
  - To minimise the potential for under-reporting would require skin photography of all main skin sites (*n* = 3 photographs covering five skin sites, sacrum, buttocks (right and left) and heels right and left) at each visit
  - To minimise the potential for over-reporting would require photography only at the point of PU development
3. Determining the ability of a photographic image to enable the assessment of early Category 2 PUs where the assessor has to be able to distinguish a small red broken skin area within a bigger area of general erythema

These issues have only been partially explored previously. We found two studies reporting the inter-rater reliability of photography assessed by different clinical experts and photography versus clinical assessment. One study compared experts’ photographic assessments, reporting levels of agreement between two groups of experts assessing photographs to classify PUs [5]. Nine trustees of the European Pressure Ulcer Advisory Panel (EPUAP) reviewed 67 photographs of normal skin, PUs of various categories and incontinence lesions. Eleven of the photographs were eliminated, as they were assessed by the EPUAP Trustees as being ‘insufficiently clear’. The 11 photographs eliminated included normal skin, non-blanching erythema and Category 2 PUs. The remaining photographs were classified by the EPUAP Trustees with 100% agreement for all photographs for eight of nine Trustees. The majority EPUAP Trustee classification was considered the ‘gold standard’. Forty-four clinical experts then assessed the photos using a web-based presentation. Areas of disagreement included classification of Category 1–4 PUs and incontinence lesions. The multirater kappa for the wider expert group was 0.80.

A further study compared the ‘gold standard’ expert clinical nurse assessment against assessment using photographs [4]. This study included 48 participants with two photographs from each: one of a PU (including Categories 1, 2, 3, 4 and unstageable) and one of an unaffected skin area. Of 32 PUs of Category ≥2 (our endpoint), sensitivity was 97% (CI 91–100%). Of the 62 pairs of photographs with Category 1 or normal skin, 60 were correctly classified with a specificity of 97% (CI 92–100%). Misclassifications were related to darkly pigmented skin. However, in translation to a potential clinical trial population, it is important to acknowledge that half the PU photographs were Category ≥3, and therefore the photographic evidence assessed is not comparable to that of ‘early’ Category 2 PUs.

Other technological solutions were also considered including laser Doppler, light spectroscopy and multispectral imaging, but these detect erythema and the intensity of skin blood flow and are unable to assess the presence of a Category 2 PU. Therefore, recognising the need to establish a method for blinded outcome and response to the funding body’s request, we set up a validation sub-study within the PRESSURE 2 trial which will address both scientific and practical questions. Therefore, recognising the need to establish a method for blinded outcome and in response to the funding body’s request, we set up a validation sub-study within the PRESSURE 2 trial which will address both scientific and practical questions.

### Objectives

The main aim of the photographic validation sub-study is to assess and quantify potential bias in the reporting of the PRESSURE 2 trial primary endpoint. The primary objectives of the sub-study are therefore to assess:

- Over-reporting of Category ≥2 PUs
- Under-reporting of Category ≥2 PUs

The secondary objectives of the sub-study are to assess:

- Rates of consent/potential impact on trial recruitment
- Acceptability to patients
- Compliance with photographs
- Compliance with secure transfer of photographs between the research site and the Leeds Institute of Clinical Trials Research (LICTR)
- Quality of photographs and confidence of photographic review
- Inter-rater reliability of photography versus clinical assessment

## Methods/design

**Fig. 1:**
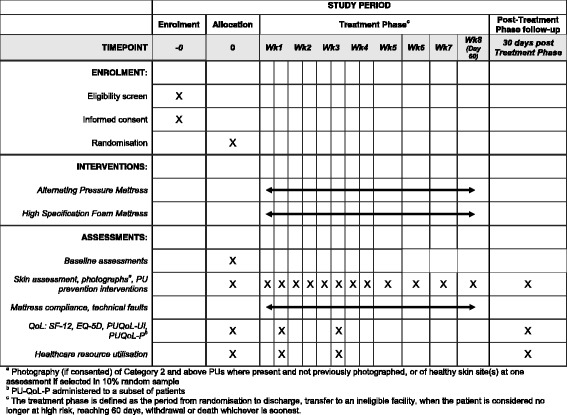
SPIRIT schedule of enrolment, interventions and assessments

**Fig. 2:**
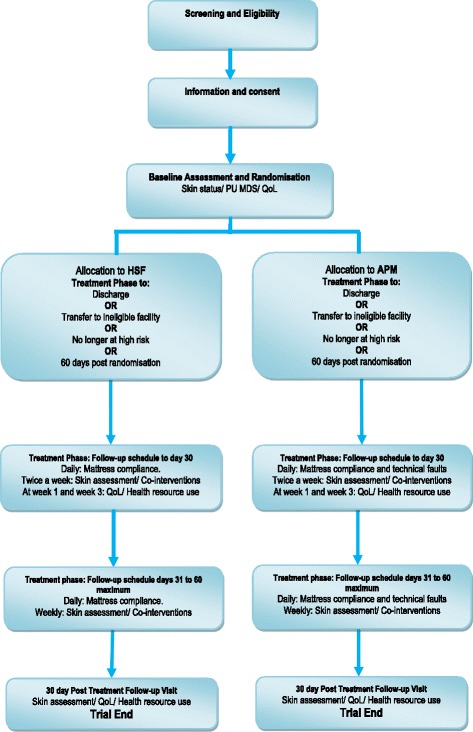
Trial flow diagram

This sub-study is being conducted within the PRESSURE 2 trial [1], which will recruit a maximum of 2954 patients who are at ‘high risk’ of PU development. The main trial is a multicentre trial, and the sub-study has aimed to reflect this through recruitment of participants from each centre. The SPIRIT flow chart and schedule of enrolement, interventions and assessments for the main trial are given here in Figs. 1 and 2 and Additional file 1.

### Patient consent

Details of the consent process for participants who have capacity and those who have cognitive impairment are given in the main protocol paper [1]. When consent to the trial is taken, patients will be specifically asked to consent to photographs being taken of their skin sites. An opt-out clause will allow patients to participate in the study without consent to photographs or to withdraw consent to photographs at any time.

### Photographic data collection

Photographs will be taken whenever a new Category ≥2 PU is identified. Additionally, the following procedures will be used.

To enable the assessment of the over-reporting of Category ≥2 or higher PUs, a trained research nurse will photograph all Category ≥2 PUs at first observation (baseline or follow-up).

To assess the potential for under-reporting PUs, we will need photographs of both ulcers and non-ulcerated skin to be taken by an independent (blinded) assessor. The LICTR will randomly identify 10% of patients from each centre. Randomisation will be conducted using a central automated randomisation system with allocation concealment at the LICTR, which will notify the Principal Investigator (or delegate) by email when a participant has been allocated to have this additional assessment. The local Principal Investigator or delegate will photograph two skin areas including one torso and one limb skin site. If the patient has any Category ≥2 PU, one will be photographed. If there is more than one Category ≥2 PU, the photographs will include one skin site with a PU and one skin site without.

To ensure photographs are obtained for 10% of those patients recruited, the trial team will monitor compliance, with photographs being returned on a regular basis, and adjust the proportion of patients selected on the 24-hour system to participate in the photography sub-study.

All photographs will be sent to the LICTR for blinded central endpoint review by a panel of independent clinical experts. Transfer of photographic data will comply with data protection legislation (details are given below). The data collection and transfer process will be monitored to assess compliance and reasons for any non-compliance.

Information from the anonymised screening logs and trial consent forms (opt-out of consent to photographs) will be used to assess rates of consent to photographs and potential impact on trial recruitment. The acceptability to patients will be assessed using data that will be collected for patients not consenting to having photographs taken and for those who do consent. Reasons why any photographs are not taken will also be collected, e.g. ‘patient unwell’.

### Sample size

In PRESSURE 2 we will be recruiting a maximum of 2954 patients with an assumed overall event rate of 20.5%. Let us assume that each patient has 3 pressure areas (as described above) photographed 8 times in total if they don’t develop a PU (photos to end of study) and 4 times if they do (assuming that we stop photographing once they have a PU). This could result in up to (2354 × 3 × 8) + (600 × 3 × 4) – 600 = 63,096 negative photos + 600 positive photos, totalling 63,696 photos. This number of photographs would clearly be a burden to staff and patients.

In light of unrealistic sample sizes and the evidence described above suggesting problems with levels of agreement between experts assessing photographs [5], as well as the absence of robust evidence relating to the accuracy of photographic skin assessment compared to the current ‘gold standard’ or the availability of technological methods of skin assessment, the first stage of a validation study is needed to address the scientific concerns. That is, a study is needed to determine the validity of photography versus the ‘gold standard’ expert nurse clinical assessment in the assessment of Category 2 PUs.

There has been no formal sample size calculation for this sub-study due to its exploratory nature. However, we can anticipate the number of photographs that are expected based on PU incidence rates from previous research and the sample size of the main trial [1]. A maximum of ~1653 photographs are expected for the central blinded review, which will enable kappa to be estimated to within a precision of at least ±0.044 (corresponding to the half width of the 95% CI), assuming 65% of photographs are of Category 2 or higher PUs and kappa ≥0.5.

To quantify the extent of over-reporting, we consider the following. Using frequency distributions of the number of Category ≥2 PUs observed at baseline and during follow-up in the pain cohort study, which recruited a similar population [6], we can expect up to 1690 new PUs to be photographed, corresponding to 803 PUs at baseline (27.2% of patients recruited) and 878 PUs that develop during follow-up (from 605 (20.5%) participants). The true number is likely to be lower due to consent rates for photography, which could change throughout the study. For example, a patient could provide consent for photographs to be taken at baseline, but at a subsequent visit he/she may not provide consent for further photographs to be taken.

To assess under-reporting, the following is considered. We expect 296 patients to be selected for this part of the study. Assuming they are assessed close to the baseline visit and assuming the proportion of patients observed to have a PU at baseline similar to that in the pain cohort study (27.2%), then we can expect 80 photographs of PUs and 512 photographs of PU-free skin sites, providing a total of 774 photographs from the independent assessment.

Therefore, a maximum of 2455 photographs corresponding to 1943 PU photographs and 512 pressure-ulcer free photographs can be expected to be received and reviewed by the central review panel.

### Camera and photographs

The expertise of an independent professional medical photographer was sought for camera selection, testing and standardisation of photographs.

#### Choice of camera

A practical consideration of the study was the cost of supplying 50 cameras to sites taking part in the study. A digital single-lens reflex (DSLR) camera with macro lens and flash could cost £1000–£3000 per unit depending on quality. Compact cameras recommended by the Institute of Medical Illustrators (IMI) National Guidelines on Wound Management [7] such as the Nikon Coolpix P7100 costing £345 per camera would cost the study £17,250. With consideration to the available budget of the study, potential affordable camera models were tested using a ColorChecker Color Rendition Chart to assess colour accuracy. The selected camera, the Canon IXUS 510 HS, was then tested further to assess the best settings and shooting distances to standardise the cameras.

The study planned to use the same model Canon IXUS 510 HS camera for more consistent results. Different camera models, even by the same manufacturer, will use different sensors, lenses and firmware. Inconsistent use of cameras could make a difference in the quality of the images, in particular if the camera had a colour bias, and therefore could affect the assessment of the PU.

#### Camera testing

The setup of the camera was considered and a guide produced to standardise the functions of the camera. The flash was set up to be the primary lighting source in all images and a fixed shooting mode recommended so that the camera would not vary in different lighting settings across all of the units involved in the study. By manually setting up the camera settings, the International Standards Organisation (ISO), exposure compensation, white balance, light metering and colour settings could all be programmed for consistent results. A shooting distance of 30 cm between the camera and skin site was recommended so that the built-in flash would not overexpose the subject area.

**Fig. 3:**
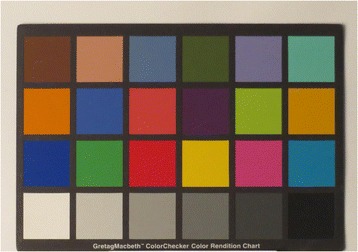
Colour accuracy out of camera: auto white balance

**Fig. 4:**
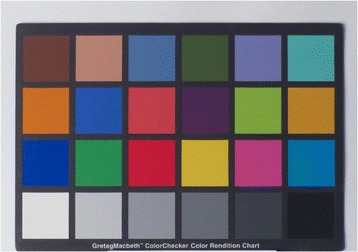
Colour accuracy out of camera: post white balance

**Fig. 5:**
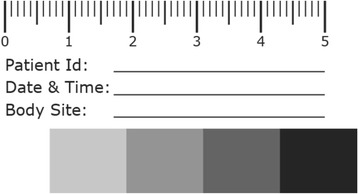
Example of grey scale card used in each photograph

Unknown variables such as unit lighting conditions were considered and a grey scale produced for inclusion in the photographs. Including a grey scale would allow LICTR personnel to process the submitted images to a more accurate colour by using a corrected white balance. By using a grey scale to correct colour during post processing, this workflow would enable the study to achieve a more consistent result in the images produced. Figures 3 and 4 demonstrate the effect of correction of white balance. See Fig. 5 for an example of the grey scale card used in every photograph.

#### Standardisation of photography technique

A study camera will be supplied to each site together with a work instruction detailing the use of a standardised photographic method including the use of grey scales for white balance and camera settings, e.g. ISO setting, etc. For consistency and interpretation of photographic data, it was deemed imperative that *only* the study camera supplied will be used to take photographs. In addition, the work instruction will provide clear instructions on the anonymisation, secure transfer and deletion of the photographs (that is, there will be no local storage of photographs on the camera or National Health Service (NHS) computer).

#### Safe transfer of photographs

To ensure the safe transfer of photographs, the LICTR will create an NHS mail email account so that sites can email photographs from their personal NHS mail email account to the study coordinating centre via a secure electronic pathway. This method of transfer for patient sensitive data is in compliance with NHS data transfer policy. During centre setup, site staff will be requested to apply for an NHS mail email account if they do not already have one. Authorised members of staff at the LICTR will be given access to the LICTR NHS mail email account and will check the account on a daily basis for new emails containing photographs. When an email containing a photograph is received, the photograph is downloaded and saved in a restricted access folder on the secure server at the LICTR. The file will be saved in the following format: Participant Trial Number_Body Site Photographed_Date of Photograph_Time of Photograph. The site will then be emailed to confirm receipt of the photograph and to request the member of staff at the site to delete the photograph from their camera, computer and email outbox. If a response from the LICTR is not received within 24 hours of the photograph being sent, the site will be requested to call the LICTR to check whether the photograph has been received. The LICTR will log each photograph and assign a unique 5-digit number in order to anonymise the photograph before it is reviewed. The LICTR will then delete the email containing the photograph from the NHS mail account and clear the downloaded file from their Internet history to ensure that the photograph is not accessible via an unsecure server.

#### Standardisation of photograph quality and independent expert review

Prior to central review by the expert clinical panel, the LICTR will ensure that the image has a corrected white balance, is cropped and is made anonymous to the reviewers. To achieve this, the photograph will be imported into the Adobe Lightroom software. The included grey scale in the image will then be used as a reference for the software to correct the white balance to a true white for the lighting conditions under which the photograph was taken. Once it has been corrected, the grey scale card is cropped out of the picture so that no participant details are visible during the clinical review. The photograph will then be saved in a separate folder on the secure server with the file name in the following format: Body Site Photographed-unique 5-digit photograph number. The photograph will then be deleted from the Adobe Lightroom software and entered as ‘Processed – Waiting for Review’ on a spreadsheet used to log the receipt of each photograph.

During the central photographic review, the reviewers will be expected to document their assessment of the skin site using the EPUAP classification scale [2]. They will also be asked to report any Category 1 pressure damage identified and classify other skin abnormalities, e.g. moisture damage, eczema, etc. It is recognised that, despite clear instructions, the photographs will not be taken by medical photographers and therefore may not be of high quality. Also, identifying Category 1 damage requires a combination of visual and manual examination of the skin, and the diagnosis of other skin conditions requires details of the clinical history. As the central reviewers will be asked to make a decision on the photograph alone, they will be asked to rate their confidence in their decision using an adapted 11-point scale ranging from 0 (Not confident at all) to 10 (Very confident) [8]. Three members of the central review panel will independently review the photographs; the assessment used for summaries and analysis will be the category for which there are at least two members of the panel in agreement.

On the day of each review, the computer monitor on which each photograph will be viewed is calibrated using the Spyder 4 Pro Advanced Monitor Calibration software to ensure that the colours in the photograph are displayed as accurately as possible on the assessment monitor. Wherever possible the same computer monitor will be used for each review to minimise the impact of variations such as ambient light in the room. During the review each member of the expert clinical panel will be asked to view the photograph on the screen one at a time and record their assessment on a trial case report form (CRF) including the Body Site Photographed and the unique 5-digit photograph number for identification purposes. A member of the LICTR will be present at each review session to ensure that the panel members do not discuss details of their assessment.

Following each review, the CRF will be ‘unblinded’ by the LICTR by linking the unique 5-digit photograph number to the correct participant number and entered into the trial database.

### Analysis

#### Assessing risk of over-reporting

A sensitivity analysis will be conducted in addition to the main trial primary analysis whereby the primary endpoint will be replaced by the primary endpoint that would have been derived if the assessment made by the blinded central endpoint review of photographs had been used. That is, for all Category ≥2 PUs that were reported by the research nurse, these assessments will be replaced with the assessments made by the blinded central endpoint review. The primary endpoint will remain the same for skin sites that were not assessed as Category ≥2 by the research nurse or for those for which photographs were not received.

#### Assessing risk of under-reporting

For each skin site assessed by the local Principal Investigator (or delegate), a 2 × 2 table will be produced to summarise whether the skin assessments made by the local Principal Investigator (or delegate) and the skin assessments made by the research nurse agree on the Category ≥2 PU status on a patient level. In addition, there will be a 2 × 2 table summarising the agreement across all skin sites.

For the skin sites photographed by the local Principal Investigator (or delegate), 2 × 2 tables will be produced for each skin site and overall to summarise the agreement on the Category ≥2 PU status for each of the following comparisons:

- Assessments drawn from the photographs (from blinded central review) and the skin assessments made by the local Principal Investigator (or delegate)
- Assessments drawn from the photographs (from blinded central review) and the skin assessments made by the research nurse, and also
- Clinical skin assessments made by the local Principal Investigator (or delegate) and the skin assessments made by the research nurse

Kappa and prevalence and bias adjusted kappa statistics will be reported for each of these tables [9, 10].

#### Rates of consent/potential impact on trial recruitment

To assess the rates of consent/potential impact on trial recruitment, the reasons for not being recruited to the main trial will be summarised to identify whether photography is preventing patients from entering the trial. This is not expected to be a reason for non-randomisation, because the photography sub-study is optional. Therefore, the consent rate for the photography sub-study will be summarised.

#### Acceptability to patients

To assess the acceptability to patients, the reasons for photographs not being attempted will be summarised and tracked over time to identify whether consent for photographs to be taken is withdrawn at a later date.

#### Compliance with photographs

Summaries of whether a photograph is expected based on the skin assessment or identification on the 24-hour system will be produced. Reasons for non-compliance (where available) will be summarised. Compliance with the protocol for taking photographs will also be summarised, such as whether the grey scale card was used.

#### Compliance with secure transfer of photographs

Summaries of any deviations from the work instruction for the secure transfer of photographs from the research site and the LICTR will be presented, together with any remedial action taken.

#### Quality of photographs and confidence of photographic review

The number of photographs considered to be unable to be assessed will be summarised together with the reasons. The confidence of the photographic assessments will be summarised overall and also where any discrepancies arise between the photographic and clinical assessments. Listings and summaries of each reviewer’s assessment of each photograph will be produced to identify whether there are any particular skin sites or category of PUs that cause difficulties when PU photographs are assessed.

## Discussion

A validation sub-study, using photography with blinded central review, will be carried out to assess any under-reporting or over-reporting of Category ≥2 PUs and establish whether future trials can utilise central review of photography for blinded primary outcome assessment.

Guidelines which have been prepared for use by professional medical illustrators exist for clinical photography of patients’ wounds [7]. This study acknowledges the application of these guidelines would be the ‘gold standard’ in photography, but the need for a pragmatic approach dictated the process used.

As per IMI National Guidelines, the use of cameras on mobile phones could not be allowed because it would introduce unacceptable risks of compromising patient privacy. Along with budget considerations, this left only the choice of consumer compact cameras as a viable option. Since the study was set up, hybrid cameras, entry-level DSLR cameras and compact system cameras (CSCs) have improved and prices lowered, although not to the same level as for the sub £100 compacts tested for the study.

The camera selected for the study, a Canon IXUS 510 HS, has a 10-megapixel complementary metal-oxide-semiconductor (CMOS) sensor with a Digic 5 processor and a 35 mm equivalent: 28-336 mm f/3.4–5.6 lens. As the images taken would need to be emailed and would be assessed on a monitor, the 10-megapixel resolution would not be a significant issue.

As the study would be utilising non-professional photographers to photograph the patients, the workflow for the study would be limited. As most photography would take place on wards over multiple sites across the country, there would be little control or consistency between backgrounds and ambient lighting. The study recommended that lighting should always be with the camera’s flash. The white balance was to be set to Auto, as the camera did not include a flash white balance setting. This was a contributing factor to the use of a basic grey scale. The auto white balance (AWB) setting would automatically set the white balance of the camera to the optimal settings of the environment. With inconsistent environmental lighting the AWB colour setting needed to be corrected to a standardised reference point.

Commercial grey scales and colour rendition charts are available. The Xrite ColorChecker Passport, a pocket-sized version with its own camera calibration software, was tested. This could be considered the ‘gold standard’ for automating colour correction and white balance of the images produced by a camera. This software was only able to calibrate and create custom Digital Negative (DNG) profiles. Consumer cameras at the budget level tested most commonly output images to a JPEG file format, not a DNG RAW file. At a cost of £80 per chart, purchasing these charts to use as a grey scale would double the setup costs for the study. A further consideration was the risk of infection. The decision was taken to produce a disposable grey scale specifically for the study. This would give a target of white balancing images as well as including vital study information.

One noteworthy issue that would be encountered during the setup of such a study is the ongoing obsolescence lifecycle of consumer technology. The Canon IXUS 510 HS was known to be at the end of its production cycle after the initial bulk purchase of cameras. This means that the new centres will have to use a different model of camera that will not be consistent with the standardisation set up for the study.

We will also be assessing the practical aspects of using photography such as acceptability to patients, potential impact on trial recruitment, compliance with taking photographs, reasons for non-compliance, whether nursing staff (amateur photographers) can produce reliable photographs given the constraints of the clinical environment and patient positions and whether photographs can be used to assess the trial endpoint.

There are ethical issues associated with photography of PUs and skin areas at ‘high risk’ of PU development (e.g. sacrum and buttocks). The use of photography was discussed at a Pressure Ulcer Research Service User Network (PURSUN) meeting. PURSUN is a group of people with personal experience of PUs who are supporting the PRESSURE 2 study. A member of PURSUN with experience in having a PU photographed also helped to develop the PRESSURE 2 protocol and photography instructions.

Input from PURSUN led to some important measures being put in place to minimise participant burden, improve recruitment and maintain dignity:

- Verbal agreement is required at the time the photograph is taken, even if written consent has already been obtained.
- Photographs are taken by a registered healthcare professional (not a medical photographer).
- The photography instructions stress that patient comfort, privacy and dignity are paramount when positioning people for photographs.
- Participants are able to opt out of the photography sub-study but still remain in the trial.

If we find that only a significant minority of patients do not find photography of body sites acceptable or that the findings of the sub-study do not agree sufficiently with the clinical review, then this will affect its utility for central blinded review and the primary outcome measure in future trials.

Consideration will be given to the clinical application of wound photographs for the assessment of wounds. With the increasing use of ‘telemedicine’ and remote clinical assessments of patients with wounds and other skin conditions, it will be important to know the confidence in the accuracy of the remote assessment.

There is very little known regarding the pathophysiology of PU development due to issues such as ethical concerns about skin and wound biopsy of these patients [11]. It is possible that in the future, if photography is deemed acceptable to patients, photographic data collected in trials such as PRESSURE 2 could be used for further analysis, e.g. informing the pathophysiological development of PUs.

Additionally, training staff on skin and wound assessment is often difficult at the bedside due to acuity of illness, poor lighting, patient position and comfort. With permissions these photographs could be used for training purposes.

PRESSURE 2 will be the first PU prevention trial, to our knowledge, to use photographic information to contribute to the primary outcome. We need to understand the acceptability of photography for prevention trials, where missing data can reduce the power and internal validity of the trial. In leg ulcer healing studies there are a small number of high-quality trials in which the outcome has been validated through photography [12, 13]. It is not currently understood whether patients will allow photography of PUs and intact skin over bony prominences such as the sacrum, buttocks and hips during trial follow-up.

### Trial status

The first patient was randomised on 14 August 2013. As of 13 April 2016, 1501 patients had been randomised. Recruitment finished on 30 November 2016 with a total of 2030 patients recruited.

## Additional file

Additional file 1: SPIRIT checklist. (DOC 122 kb)

## Abbreviations

APM: Alternating pressure mattress
AWB: Auto white balance (camera)
DSLR: Digital single-lens reflex (camera)
EPUAP: European Pressure Ulcer Advisory Panel
HSF: High specification foam
ISO: International Standards Organisation (camera)
LICTR: Leeds Institute of Clinical Trials Research
NHS: National Health Service
NICE: National Institute for Health and Care Excellence
PU: Pressure ulcer
PURSUN: Pressure Ulcer Research Service User Network
